# Effect of lentivirus-mediated CFTR overexpression on oxidative stress injury and inflammatory response in the lung tissue of COPD mouse model

**DOI:** 10.1042/BSR20193667

**Published:** 2020-01-29

**Authors:** Xiaoli Xu, Huimin Huang, Xiangyi Yin, Hongmei Fang, Xiaoyue Shen

**Affiliations:** Department of Infection Control, Jinling Hospital, Medical School of Nanjing University, Nanjing 210002, Jiangsu Province, China

**Keywords:** chronic obstructive pulmonary disease, cystic fibrosis transmembrane conductance regulator, inflammatory injury, oxidative stress, signal pathway

## Abstract

We aimed to investigate the regulatory mechanism of lentivirus-mediated overexpression of cystic fibrosis transmembrane conductance regulator (CFTR) in oxidative stress injury and inflammatory response in the lung tissue of mouse model of chronic obstructive pulmonary disease (COPD). COPD mouse model induced by cigarette smoke was established and normal mice were used as control. The mice were assigned into a normal group (control), a model group (untreated), an oe-CFTR group (injection of lentivirus overexpressing CFTR), and an oe-NC group (negative control, injection of lentivirus expressing irrelevant sequences). Compared with the oe-NC group, the oe-CFTR group had higher CFTR expression and a better recovery of pulmonary function. CFTR overexpression could inhibit the pulmonary endothelial cell apoptosis, reduce the levels of glutathione (GSH), reactive oxygen species (ROS), and malondialdehyde (MDA) and increase the values of superoxide dismutase (SOD), GSH peroxidase (GSH-Px), and total antioxidant capacity (T-AOC). The overexpression also led to reductions in the white blood cell (WBC) count in alveolus pulmonis, the concentrations of C-reactive protein (CRP), interleukin (IL)-6, and tumor necrosis factor-α, and the protein expressions of NF-κB p65, ERK, JNK, p-EPK, and p-JNK related to MAPK/NF-κB p65 signaling pathway. In conclusion, CFTR overexpression can protect lung tissues from injuries caused by oxidative stress and inflammatory response in COPD mouse model. The mechanism behind this may be related to the suppression of MAPK/NF-κB p65 signaling pathway.

## Introduction

Chronic obstructive pulmonary disease (COPD) is a type of chronic lung disease characterized by persistent and incomplete reversible airflow restriction in airway and slow degeneration in pulmonary function [1,2]. Clinical manifestations of COPD include respiratory muscle atrophy and decreased contractility, endurance, and defense function of the lung [3]. COPD has both high incidence and mortality which have been rising each year. World Health Organization has even predicted that COPD will become the fourth leading cause of death by 2030 [4].

Causes of COPD remain unclear. It is generally believed that the disease is the result of actions by both environmental (extrinsic) factors and hereditary (intrinsic) factors. Environmental (extrinsic) factors mainly include smoking, inhalation of dust and chemicals, air pollution, respiratory tract infection, and exposure to biological dye. Hereditary (intrinsic) factors include lack of α1-antitrypsin and aberrant gene expressions of glutathione (GSH) S-transferase gene, and interleukin (IL)-10. Oxidative stress is one of the key components in the pathogenesis of COPD. It can damage the lung tissue directly and cause oxidative inactivation of protease, exudation of inflammatory cells, and gene expression of proinflammatory mediator, thereby promoting the occurrence and development of COPD. GSH, reactive oxygen species (ROS), superoxide dismutase (SOD), and GSH peroxidase (GSH-Px) are all markers in oxidative stress. Moreover, the inflammatory response in COPD is a type of oxidative reaction in the airway area and is the manifestation of the acute exacerbation of COPD [5].

In recent years, cystic fibrosis transmembrane conductance regulator (CFTR) as a drug target in some major diseases has been gaining increasing attention. The latest clinical research has found that the expression of the ion channel protein CFTR can decrease significantly in patients with COPD [6]. This finding indicates that CFTR may be related to the pathogenesis of COPD. CFTR belongs to the ATP-binding cassette family of membrane transport proteins. It is a type of Cl^−^ channel in epithelial cell regulated by phosphorylation and is expressed in many apical membranes of epithelium related to fluid secretion and absorption [7]. CFTR, as a chloride channel activated by cAMP, participates in the secretion and transport of transepithelial ion and water molecule in endothelial cells through Cl^−^ transmembrane transport and serves a critical role in the cell proliferation, migration, and ion concentration adjustment [8].

Some studies have reported that malfunction in CFTR can not only bring about the inherited disease, cystic fibrosis, but is also closely associated with chronic idiopathic pancreatitis, male infertility, and pancreatic dysfunction. COPD is regarded as the most severe symptom of CFTR malfunction [9,10]. Thus, we speculated that CFTR might be a potential treatment target in the novel therapy for COPD.

## Materials and methods

### Experimental animals

Forty healthy male Kunming mice (21 ± 3 g, 6–8 weeks old) were chosen for the study. The mice which were specific-pathogen-free were provided by Guangxi Medical University Laboratory Animal Center (animal permit number: SCXK, Guijian, 2013-0016). According to the random number table, 10 mice were assigned to a normal group as control, whereas the rest 30 mice were used for COPD modeling. The protocol for the research project has been approved by the Ethics Committee of Jinling Hospital, Medical School of Nanjing University (Approval No. 20180016). The experimental research with animals was conducted in the Laboratory of Department of Infection Control, Jinling Hospital, Medical School of Nanjing University.

### Establishment of COPD mouse model and grouping of subjects

COPD mouse model was induced by smoke exposure. The mice were placed in a smoke exposure box with two holes (1.5 cm × 1.5 cm) at the top of the box to prevent hypoxia. Next, cigarettes removed off filter tips were lit and inserted to the box, and the box was closed. Nine cigarettes were lit each time. After 1 h, mice were moved out of the box for a 15-min break before going through the above steps again. Two rounds of smoke fumigation were performed in the morning, and another two rounds were performed after an interval of 4 h. The steps above were carried out for six consecutive days every week, lasting 24 weeks. During the process of fumigation, all mice were weighed every other month. After fumigation ended, the COPD modeling and normal mice were fed normally under the same condition, and markers regarding pulmonary function such as peak inspiratory flow (PIF), peak expiratory flow (PEF), inspiratory resistance (RI), and dynamic lung compliance (Cdyn) were measured to assess the modeling results.

The successfully modeled mice were randomized into the following groups of ten mice each: model group (untreated), oe-NC group (intravenous injection of 40 μg negative control vector), and oe-CFTR group (intravenous injection of 40 μg CFTR overexpression vector). Pulmonary function in each group was examined after 1 week of injection.

### Evaluation of mice’s pulmonary function

After smoking, intraperitoneal injection of 100 mg/kg, 1% pentobarbital sodium was administered in each group for anesthesia, and tracheostomy was performed at the thyroid area with an incision as small as possible. Following intubation, mice were placed in a plethysmograph, and a small animal ventilator was connected for mechanical ventilation (Harvard Apparatus, Inspira, The United States). The ventilation parameters were set as follows: 10 ml/kg tidal volume, 8–12 cm H_2_O intake pressure, 120 beats/min respiratory rate, and 10:15 inspiratory–expiratory ratio. After mice could fully coordinate breathing with the ventilator, their pulmonary ventilation performance was examined (Buxco, PFTManeuvres, The United States), and levels of PIF, PEF, RI, and Cdyn were measured in each group.

### Construction of lentiviral vectors overexpressing CFTR

The mouse CFTR gene sequence was obtained from GenBank. After total RNA extraction, RNA samples were reverse transcribed into cDNAs using PrimeScript™ RT reagent Kit (Takara, China) according to the manufacturer’s instructions. The cDNAs were used as templates, and target fragments were obtained after PCR amplification. Double enzyme digestions with EcoR I and Xho I were conducted in pcmv-myc vector. Fragments of linearized vectors were collected and linked to target fragments using Solution I (Dongsheng Biotech, China). After linking at 16°C for 8 h, the recombinant plasmid was transfected into DH5α competent cell. The positive monoclonal colony was then selected following coating and cultured for amplification. The plasmid was extracted using endotoxin-free plasmid maxiprep kit (Merck, Germany). The plasmid was transfected into 293T cell and cultured for 48 h, and the supernatant was taken out before lentivirus packaging. Viral titer was measured, and liposome was obtained. Afterward, intravenous injection of the packaged liposome-encapsulated recombinant plasmid overexpressing CFTR and the liposome-encapsulated recombinant plasmid with negative expression were administered to mice (dose: 40 μg liposome-encapsulated recombinant plasmid to each mouse through tail vein, three times per day for 1 week). The CFTR expression levels were then examined by qRT-PCR in the normal, oe-NC, and oe-CFTR groups.

### Bronchoalveolar lavage fluid collection and white blood cell count measurement

After measuring the pulmonary function in each group, the skin and muscles at the neck and chest area of mice were incised in the oe-NC and oe-CFTR groups to expose bronchus and lung. The right bronchus was clipped, and a tracheal trocar was inserted into the left bronchus. Left lung lavage was then performed for three times with 9 ml normal saline in a 10-ml external syringe. The bronchoalveolar lavage fluid (BALF) was collected and filtered with gauze. White blood cell (WBC) was counted on a blood cell counting plate using 0.1 ml filtered BALF, and the counting was repeated three times independently.

### Hematoxylin and Eosin stain

Right upper lobes in each group were collected for making paraffin sections. The section was fixed with 4% paraformaldehyde for 24 h and dehydrated in a gradient of alcohol (80–100%) and normal butyl alcohol. Next, samples were placed in a wax box for paraffin embedment at 60°C and sectioned. The sections were flattened at 45°C and picked up and dried at 60°C for 1 h followed by dewaxing in xylene. Afterward, the samples underwent hydration through a gradient of alcohol. The sections were stained with Hematoxylin for 2 min and rinsed in double distilled (dd) water for 10 s. Hydrochloric acid-alcohol (1%) was applied for differentiation for 10 s followed by washing in dd water for 1 min. Subsequently, sections were stained with Eosin for 1 min and rinsed in distilled water for 10 s before dehydration through gradient alcohol (80–100%). The sections were then cleared in xylene and sealed in neutral balsam followed by being observed under a light microscope (XP-330, Shanghai Bingyu Optical Instrument Co., Ltd., China) to detect the pathological changes in bronchus and lung tissues.

### TUNEL stain

Right upper lobes in each group were collected for making paraffin sections. The samples were immersed and washed in xylene twice for dewaxing (5 min each time) and hydrated in a gradient of alcohol (100–70%, 3 min each time). Next, the samples were treated with proteinase K for 30 min at 37°C and washed in PBS twice (10 min per wash). Slides were wiped dry, and 50 μl TUNEL reaction mixture was added to the sample. After sealing with a sealing film, samples were incubated in a dark wet box for 1 h at 37°C followed by washing in PBS for three times (10 min per wash). Slides were wiped dry, and samples were added with 50 μl Converter-POD. After applying the sealing film, samples were placed in a dark wet box again for 1 h at 37°C followed by three washes in PBS (10 min per wash). Next, samples were treated with 50 μl DAB substrate in a dark wet box for 1 h at 37°C and washed in PBS for three times. Subsequently, sections were dehydrated with gradient alcohol (80–100%), cleared in xylene, and sealed with neutral balsam. Apoptosis of pulmonary endothelial cells was then examined under an electron microscope.

### Assessment of oxidative stress injury

Left upper lobes (200 mg) in each group were collected for making 10% tissue homogenate. The supernatant was obtained following centrifugation and values of ROS, GSH, GSH-Px, SOD, malondialdehyde (MDA), and total antioxidant capacity (T-AOC) were measured. All kits were purchased from Shanghai Enzyme-linked Biotechnology Co., Ltd.

### Levels of inflammatory factors measured by ELISA

Left upper lobes in each group were collected for making tissue homogenate. The experiment was carried out in compliance with the manufacturer’s instructions of ELISA kit (eBioscience, Thermo Fisher, U.S.A.). The ELISA kit was first balanced under room temperature for 20 min for preparing washing solution. The standard samples were dissolved and added to the reaction plate (100 μl) for making a standard curve. Next, 100 μl samples were added to the reaction well for a 90-min incubation at 37°C. After washing, samples were treated with 100 μl biotin antibody working fluid for 60 min at 37°C followed by washing and being kept away from light. Subsequently, samples were incubated with 100 μl enzyme binding reagent for 30 min at 37°C. The plate was washed for three times and added with 100 μl substrate and incubation in the dark for 15 min at 37°C. The stop solution was quickly added to stop the reaction. Optical density (OD) value at 450 nm was measured using a multi-mode microplate reader (BioTek Synergy™ 2, U.S.A.). The standard curve was plotted based on the OD value and levels of TNF-α (Abcam, U.K.), IL-6 (Abcam, U.K.), and C-reactive protein (CRP, Abcam, U.K.) in each group were measured.

### qRT-PCR

Lung tissues in each group were collected for preparing tissue homogenate. The total RNA was extracted by TRIzol, and its purity and concentration were measured. Next, RNA samples were reverse transcribed into cDNAs using PrimeScript™ RT reagent Kit (Takara, China) with a total reaction volume of 20 μl. The cDNA samples in each group were collected, and qPCR was conducted using SYBR® Premix ExTaq™ II kit (Takara, China) according to the manufacturer’s instructions. The total reaction volume was 20 μl, including 2 μl cDNA template, 1 μl PCR forward primer, 1 μl reverse primer, 1 μl ROX Reference dye (50×), 10 μl SYBR® Premix Ex Taq™ II (2×), and 5 μl dd H_2_O. The running parameters for qPCR were set as follows: 95°C for 4 min (pre-denaturation), 94°C for 30 s (denaturation), and 60°C for 30 s (anneal) for 35 cycles. After adding melting curves, samples underwent extension at 72°C for 7 min. GAPDH was used as an internal control. The primer sequences, which were designed and synthesized by Wuhan Bojie Biomedical Science and Technology Co., Ltd., were listed in Table 1. Expression level was calculated by 2^−ΔΔ*C*_t_^ (ΔΔ*C*_t_ = Δ*C*_t__the experimental group_ − Δ*C*_t__the control group_, Δ*C*_t_ = *C*_t__target gene_ − *C*_t__GAPDH_). *C*_t_ represents the number of amplification cycles for the fluorescence intensity to reach the threshold after each sample was amplified. The experiment was run in triplicate independently.

**Table 1:** qRT-PCR primer sequences

	Sequence
CFTR	Forward: 5′-CCCTTCGGCGATGCTTTTTC-3′
	Reverse: 5′-AAGCCTATGCCAAGGTAAATGG-3′
GAPDH	Forward: 5′-AGGTCGGTGTGAACGGATTTG-3′
	Reverse: 5′-GGGGTCGTTGATGGCAACA-3′

### Western blot

Lung tissues in each group were collected. Homogenate was prepared using radio-immunoprecipitation assay lysis buffer containing phenylmethylsulfonyl fluoride, and the protein isolation was carried out on ice. The total protein was extracted and incubated on ice at 4°C for 30 min, and the sample was then centrifuged at 12000 rpm for 10 min. After obtaining the supernatant, the total protein concentration was measured using a bicinchoninic acid kit. The supernatant (80 μl) was collected and mixed with 20 μl of 5× SDS sample loading buffer followed by a boiling water bath for 10 min. After a short centrifugation, 10 μl supernatant was collected for SDS/PAGE, and protein samples were transferred to nitrocellulose membranes by tankblot and were blocked in 10% skim milk for 1.5 h. After rinsing in TBST, samples were incubated with primary antibodies for 2 h at 37°C, which were rabbit polyclonal antibody anti-CFTR (1:1000, Ab 181782, Abcam, U.K.), p-ERK (1:500, Abcam, U.K.), GAPDH (1:2500, Abcam, U.K.), rabbit anti-mouse NF-κB p65 (1:50000, Abcam, U.K.), ERK (1:1000, Abcam, U.K.), JNK (1:1000, Abcam, U.K.), and p-JNK (1:10000, Abcam, U.K.). After washing in TBST for three times (5 min per wash), samples were incubated with goat anti-rabbit IgG secondary antibody (1:2000, Abcam, U.K.) for 1.5 h at 37°C followed by three washes in TBST (5 min per wash). Subsequently, the membrane was treated with the developer and placed in a gel imager. The image was visualized and photographed by Bio-Rad image analysis system (ChemiDoc MP, Bio-Rad, U.S.A.). Protein band grayscale was analyzed with Quantity One v4.6.2 software, and protein content was calculated as protein band grayscale/internal control band grayscale. The experiment was run in triplicate independently.

### Statistical analysis

Statistical software SPSS 21.0 (Chicago, U.S.A.) was applied for data analysis. Measurement data are expressed as mean ± standard deviation, and comparison between two groups was conducted by one-way analysis of variance and Bonferroni post hoc test. *P*<0.05 was considered to indicate a statistically significant difference.

## Results

### COPD model was created successfully

**Figure 1:**
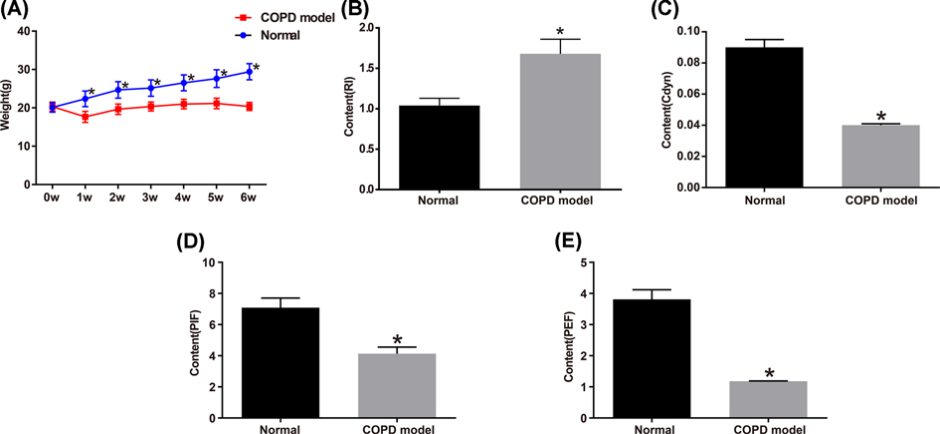
COPD model was established successfully (**A**) Weight changes in the normal group and the COPD model groups. (**B**) RI value in the normal and the COPD model groups. (**C**) Cdyn level in the normal and the COPD model groups. (**D**) PIF level in the normal and the COPD model groups. (**E**) PEF level in the normal and the COPD model groups. *P<0.05 vs. the normal group.

We compared the mice in the normal group with those in the COPD model groups and found that in the normal group, the mice were active and behaved normally; they had glossy hair with normal color, normal weight gain, and steady breathing; in contrast, mice in the COPD model groups acted slowly and lied in a curled-up posture; they had yellowish, lusterless, and messy hair and decreased appetite. Before the model creation, the body weight did not differ between the normal and the COPD model groups. However, after modeling, the weight of mice in the normal group increased steadily, whereas the mice in the COPD model groups exhibited fatigue, decreased appetite, and loss of weight in the 1st month of smoke fumigation. It was not until 1 month later that the body weight in these groups began to rise slowly, and in the 6th month after modeling, their weight was much lower than that in the normal group. Moreover, mice used for modeling had higher RI level and lower Cdyn, PIF, and PEF levels compared with the normal group (all *P*<0.05). See Figure 1.

### CFTR overexpression in mice was established successfully

**Figure 2:**
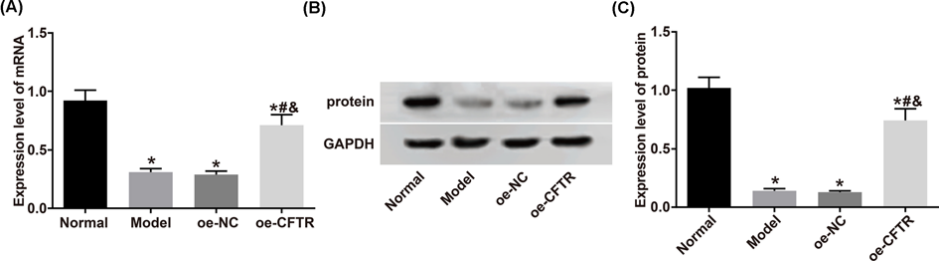
CFTR mRNA and protein expressions in each group (**A**) CFTR mRNA expression measured by qRT-PCR. (**B**) Protein band. (**C**) CFTR protein expression detected by Western blot. *P<0.05 vs. the normal group; ^#^*P*<0.05 vs. the model group; ^&^*P*<0.05 vs. the oe-NC group.

We performed qRT-PCR and Western blot to measure CFTR mRNA and protein expression levels in the lung tissues of mice in the normal, model, oe-NC, and oe-CFTR groups. The results showed that, compared with the normal group, the rest groups had much lower expression levels of CFTR mRNA and protein. Meanwhile, compared with the oe-NC group, the expression levels of CFTR mRNA and protein were higher in the oe-CFTR group (all *P*<0.05). See Figure 2.

### CFTR overexpression improved pulmonary function in COPD mouse model

**Figure 3:**
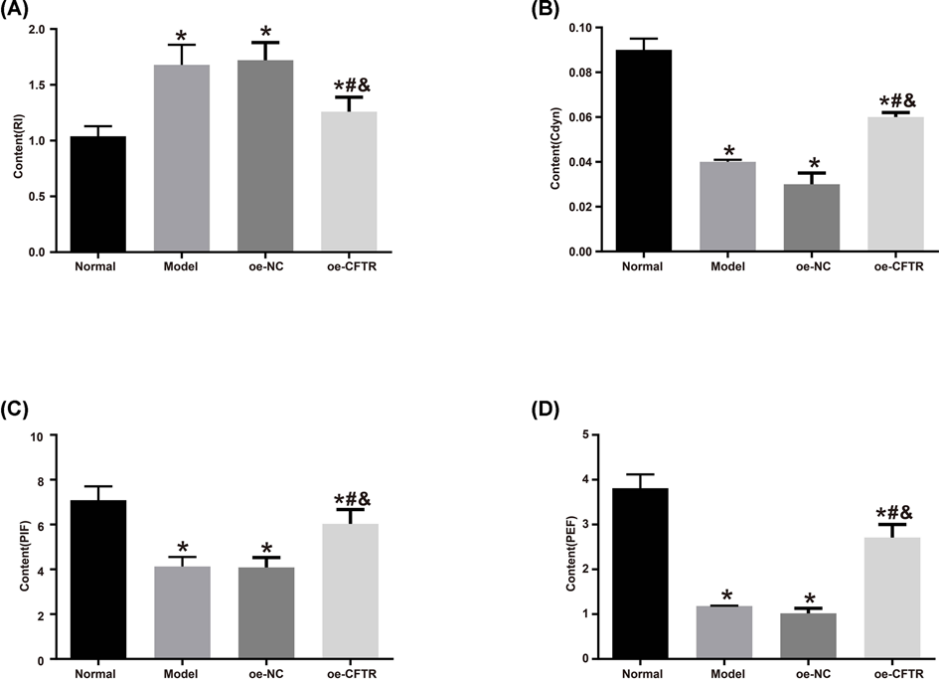
Pulmonary function in each group (**A**) RI value in each group. (**B**) Cdyn value in each group. (**C**) PIF value in each group. (**D**) PEF value in each group. *P<0.05 vs. the normal group; ^#^*P*<0.05 vs. the model group; ^&^*P*<0.05 vs. the oe-NC group.

In order to measure the effect of CFTR overexpression on pulmonary function in COPD mice, we measured the RI, Cdyn, PIF, and PEF levels in the four groups, which showed that compared with the normal group, the rest groups had lower levels of Cdyn, PIF, and PEF and a higher level of RI (all *P*<0.05). Meanwhile, compared with the oe-NC group, the level of RI was lower, and the levels of Cdyn, PIF, and PEF were greater in the oe-CFTR group (all *P*<0.05). See Figure 3.

### Histopathological changes in each group

**Figure 4:**
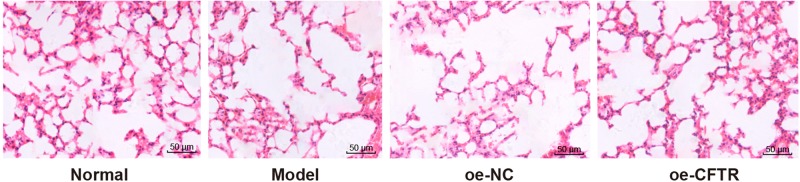
HE stain results in lung tissues of each group (200×)

Hematoxylin and Eosin (HE) staining was used to detect histopathological changes in mice’ lung tissues in the four groups. In the normal group, the epithelial cells were arranged neatly; no evident inflammatory cell infiltration was observed in the lung; the size of smooth muscle was not increased, and the alveolus had intact structure and was uniform in size. In the model and oe-NC groups, we observed partial detachment of epithelial cells, severe inflammatory cell infiltration in the lung, hyperplasia of the smooth muscle cell, increased alveolar space, thinning of the alveolar wall, and lesions in alveolus pulmonis. Compared with the oe-NC group, the oe-CFTR group had less severe pathological injury and relatively less inflammatory cell infiltration. Meanwhile, this group had thinner alveolar wall versus the normal group, increased alveolar space, and less severe fusion and injury. See Figure 4.

### CFTR overexpression reduced apoptosis of endothelial cells in lung tissues

**Figure 5:**
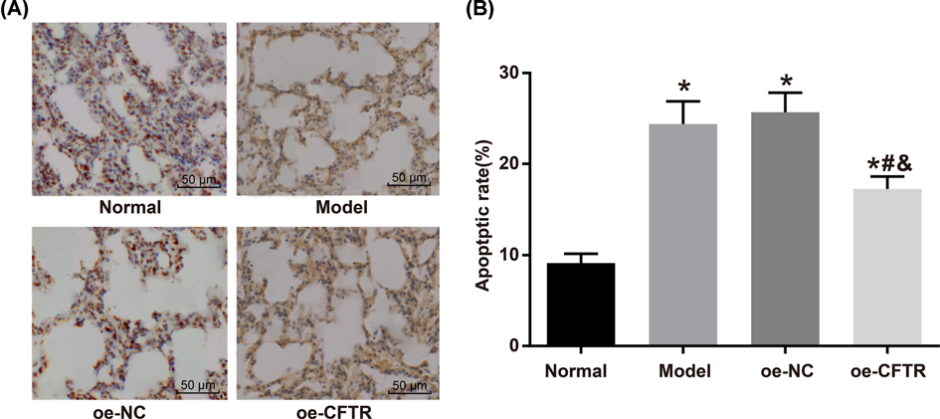
Apoptosis of endothelial cells in lung tissues of each group (**A**) TUNEL stain results (200×). (**B**) Apoptosis rate of endothelial cells in each group. *P<0.05 vs. the normal group; ^#^*P*<0.05 vs. the model group; ^&^*P*<0.05 vs. the oe-NC group.

We measured the apoptosis rate of endothelial cells in the lung tissues in the normal, model, oe-NC, and oe-CFTR groups using TUNEL stain, and the results were (9.14 ± 1.02)%, (24.39 ± 2.51)%, (25.68 ± 2.16)%, and (17.24 ± 1.36)% respectively. Compared with the normal group, the apoptosis rate of endothelial cells in the other groups rose markedly (all *P*<0.05). Meanwhile, the apoptosis rate in the oe-CFTR group was lower than in the oe-NC group (*P*<0.05). See Figure 5.

### CFTR overexpression attenuated oxidative stress injury in lung tissue

**Figure 6:**
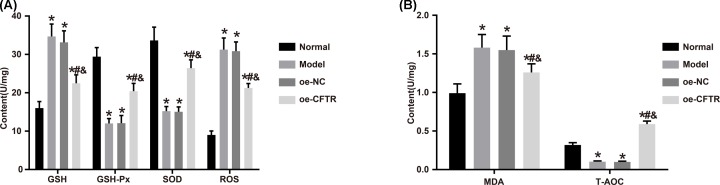
Measurement of markers regarding oxidative stress injury in each group (**A**) Levels of GSH, GSH-Px, SOD, and ROS in lung tissues of each group. (**B**) Levels of MDA and T-AOC in lung tissues of each group. *P<0.05 vs. the normal group; ^#^*P*<0.05 vs. the model group; ^&^*P*<0.05 vs. the oe-NC group.

We measured GSH, GSH-Px, SOD, ROS, MDA, and T-AOC levels in the four groups using colorimetry, and the results revealed that compared with the normal group, the other groups had higher levels of GSH, ROS, and MDA and lower levels of GSH-Px, SOD, and T-AOC (all *P*<0.05). Compared with the oe-NC group, the GSH, ROS, and MDA levels were lower and the levels of GSH-Px, SOD, and T-AOC were higher in the oe-CFTR group (all *P*<0.05). All these findings exhibited that CFTR overexpression can attenuate oxidative stress injury in COPD mice. See Figure 6.

### CFTR overexpression reduced WBC count in BALF of COPD mouse model

**Figure 7:**
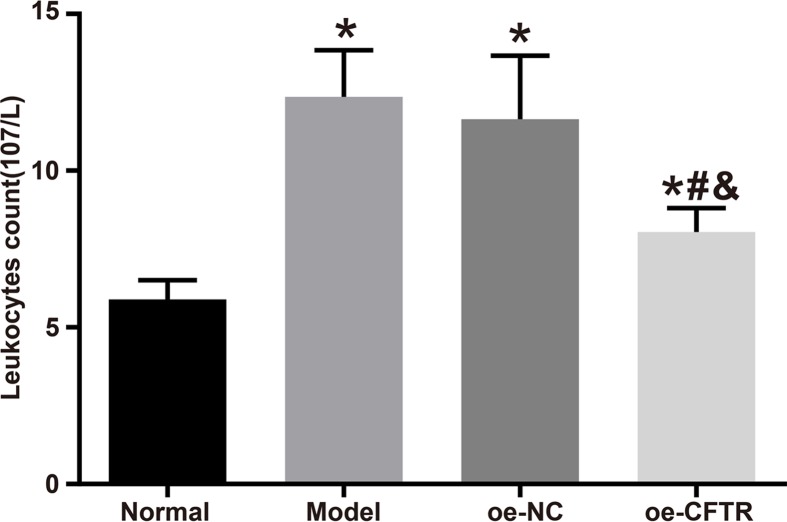
WBC count in BALF in each group *P<0.05 vs. the normal group; ^#^*P*<0.05 vs. the model group; ^&^*P*<0.05 vs. the oe-NC group.

In order to examine whether CFTR overexpression could improve inflammatory response in COPD mice, we collected BALF from the four groups to calculate WBC count. The WBC counts in the normal, model, oe-NC, and oe-CFTR groups were (5.89 ± 0.61) × 10^7^/l, (12.36 ± 1.49) × 10^7^/l, (11.64 ± 2.03) × 10^7^/l, and (8.04 ± 0.58) × 10^7^/l, respectively. Compared with the normal group, the other groups had higher WBC count (all *P*<0.05). Moreover, the oe-CFTR group had lower WBC count than the oe-NC group (*P*<0.05). See Figure 7.

### CFTR overexpression inhibited inflammatory response in lung

**Figure 8:**
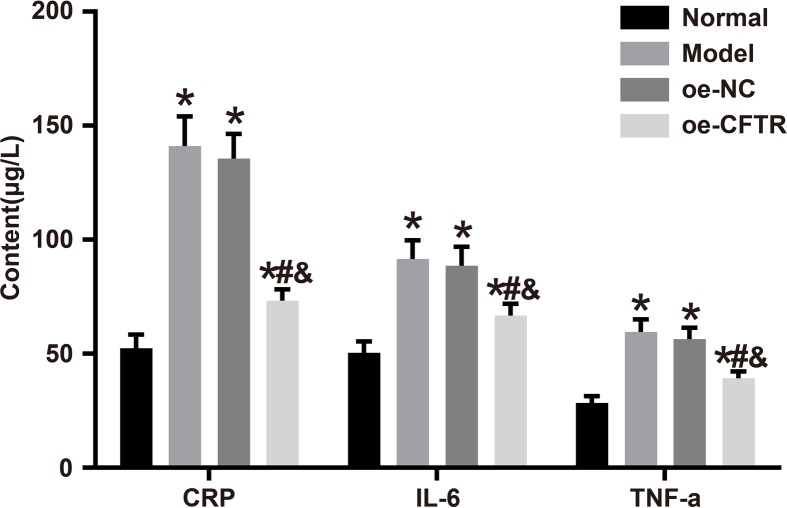
Levels of inflammatory factors in each group measured by ELISA *P<0.05 vs. the normal group; ^#^*P*<0.05 vs. the model group; ^&^*P*<0.05 vs. the oe-NC group. Abbreviation: TNF-α, tumor necrosis factor-α.

CFTR overexpression suppressed the abnormal increase in WBC count in alveolus pulmonis of COPD mice, indicating that CFTR overexpression could regulate inflammatory response in the lung of COPD mice. In the present study, we measured CRP, IL-6, and TNF-α levels in the lung tissues and found that compared with the normal group, rest of the groups had higher levels of these markers. Moreover, compared with the oe-NC group, the oe-CFTR group had lower values of CRP, IL-6, and TNF-α (all *P*<0.05). The results demonstrated that CFTR overexpression can inhibit the secretion of inflammatory factors. See Figure 8.

### CFTR overexpression inhibited oxidative stress and inflammatory response in the lung through suppressing MAPK/NF-κB p65 signaling pathway activity

**Figure 9:**
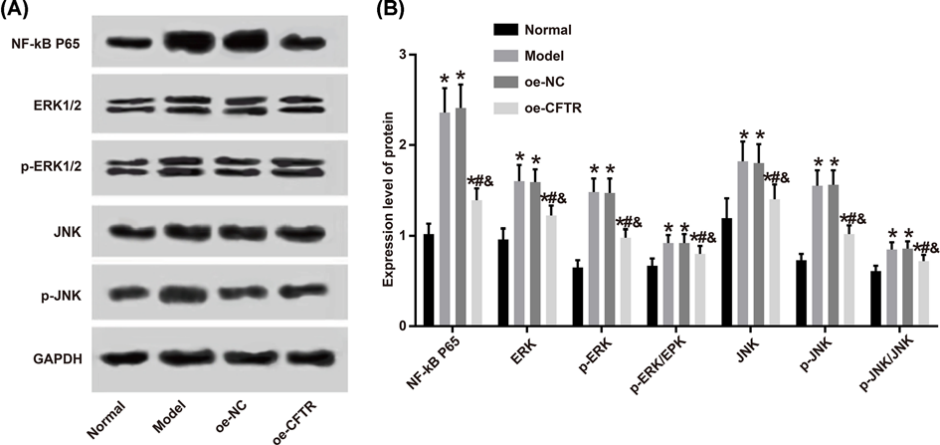
Protein expression levels in lung tissues of each group detected by Western blot (**A**) Protein band. (**B**) Expression level of protein. *P<0.05 vs. the normal group; ^#^*P*<0.05 vs. the model group; ^&^*P*<0.05 vs. the oe-NC group.

Some studies have indicated that CFTR can inhibit MAPK/NF-κB p65 signaling pathway activity, thereby suppressing the oxidative stress and inflammatory response. In the present study, we measured the protein expressions of NF-κB p65, ERK, JNK, p-EPK, and p-JNK in the four groups by Western blot and found that the levels of these proteins were elevated in the model, oe-NC, and oe-CFTR groups versus the normal group (all *P*<0.05). Meanwhile, compared with oe-NC group, oe-CFTR group had lower levels of these proteins in the lung tissues of mice (all *P*<0.05). These results demonstrated that CFTR may inhibit oxidative stress and inflammatory response in the lung through suppressing MAPK/NF-κB p65 signaling pathway activity. See Figure 9.

## Discussion

According to the statistics, approximately 600 million people are suffering from COPD worldwide, and the burden of COPD will rank fifth in global burden of disease by 2020 [11]. COPD can not only affect patients’ working ability and quality of life but can also pose a substantial financial burden on patients and society. In China, it is estimated that the incidence of COPD had reached 9.4%, revealing that the disease has high prevalence and mortality and takes much social resources within the country [12]. Therefore, it is pressing to find out an effective treatment method for this disease.

Oxidative stress is believed to be one of the essential factors in the occurrence and development of COPD. The oxidative stress reaction refers to the imbalance between the normal ratio of oxidant to antioxidant resulting from an excess of oxidants and a shortage of antioxidants in the human body, leading to tissue damage [13]. The oxidative stress increases in COPD patients as their lungs expereince long-term exposure to intrinsic or extrinsic oxidants and they have an imbalance between the oxidant and antioxidant levels due to reductions in antioxidase activity and non-enzymatic antioxidant level in the body. Some studies have reported that CFTR malfunction can influence the antioxidant system, elevate ROS markedly, and cause oxidative stress reaction [14]. Moreover, it was documented that in mice with CFTR knockout, there were a marked elevation in ROS level in the lung, reductions in the GSH level in mucous of respiratory tract epithelium and mitochondrion, and noticeable oxidative stress in mitochondrion [15]. Some studies have revealed that CFTR is the key regulatory factor in the extracellular level of GSH. It mainly exists in the endothelium of the airway and works with oxidoreductase in airway as a protective barrier [16]. GSH, as an essential antioxidant in the mitochondrion, participates in the transport between mitochondrial intermembrane space and mitochondrial matrix through dicarboxylate and oxoglutarate transporters. During oxidative stress, GSH serves the role in keeping the integrity of epithelium. Both *in-vitro* cell experiments and *in-vivo* studies in mice lungs have documented that the reduction in GSH can lead to increased permeability of epithelium. Our results also found that in the oe-CFTR group, there were much lowered GSH and ROS values, as well as increased GSH-Px and SOD levels in the lung tissues of mice versus the oe-NC group, exhibiting that the CFTR overexpression can attenuate oxidative stress injury in COPD mice.

Furthermore, we found that compared with the oe-NC group, the levels of CRP, IL-6, and TNF-α were much lower in the oe-CFTR group, suggesting that CFTR overexpression can inhibit the secretion of inflammatory factors. Since COPD is a type of airway inflammation in essence and there is an interaction and synergy between oxidative stress and airway inflammation, anti-inflammatory therapy can also be critical in the treatment of COPD. There have been studies comparing people with normal pulmonary function with patients presenting with pulmonary emphysema. Both groups had the same amount of smoking. However, the levels of macrophage, T cell, neutrophil, and eosinophil in the lungs of patients with pulmonary emphysema were increased multiple-folds compared with the normal group. This finding suggests that the inflammatory response toward smoke is elevated in patients with pulmonary emphysema [17]. In the studies using mouse models exposed to chronic cigarette smoke, amplified inflammatory cells including phagocyte, neutrophil, and T cell were observed in lung, and these cells and mediators were found to participate in the COPD chronic inflammation and affect the balance between oxidation and antioxidation [18,19]. Neutrophil, as one of the vital components in non-specific cellular immunity in the human body, is an important inflammatory cell in COPD. When neutrophil has hyperactivity in metabolism due to various factors, the metabolites including ROS radical group rises and cause inflammatory injuries in airway and lung tissues [20,21]. In studies investigating the relationship between neutrophil apoptosis delay and pathogenesis of COPD, it was found that aberrant protein expression of apoptosis family is one of the key reasons for neutrophil apoptosis [22,23]. The CFTR chloride ion serves a critical role in apoptosis through regulating the levels of ROS and GSH [24,25]. Neutrophil apoptosis delay is believed to be one of the leading causes of lung and airway chronic inflammation, and abnormality in the apoptosis regulation can sustain the chronic inflammation, thereby affecting the occurrence and development of COPD.

Pathogenesis of COPD is the result of actions and interactions of various factors [26]. As researchers are digging deeper into the study, knowledge of COPD will increase in the medical field. The present study aimed to provide some new insights into the prevention and treatment of COPD through investigating the effect of CFTR overexpression on oxidative stress and inflammatory response in lung tissues of COPD mouse model. However, gene therapy is still facing some challenges that are yet to be overcome, for example, how to choose the right target cell, how to obtain the durability of gene transfer and stability of gene integration, and how to avoid the immune response in the human body. In the present study, we did not carry out a long-term and multi-time point measurement, which is a deficiency of our study. In addition, what we used in the present study was qRT-PCR, but not TaqMan RT-PCR, a possible more accurate technology, and the two technologies may bring about some differences. It is also a deficiency of our study. We will present more comprehensive data results in deeper studies in the future. In conclusion, CFTR overexpression can protect lung tissues from injuries caused by oxidative stress and inflammatory response in COPD mouse model. The mechanism behind this may be related to the suppression of MAPK/NF-κB p65 signaling pathway.

## Abbreviations

BALF: bronchoalveolar lavage fluid
Cdyn: dynamic lung compliance
CFTR: cystic fibrosis transmembrane conductance regulator
COPD: chronic obstructive pulmonary disease
CRP: C-reactive protein
dd: double distilled
GSH: glutathione
GSH-Px: GSH peroxidase
IL: interleukin
MDA: malondialdehyde
OD: optical density
PEF: peak expiratory flow
PIF: peak inspiratory flow
RI: inspiratory resistance
ROS: reactive oxygen species
SOD: superoxide dismutase
T-AOC: total antioxidant capacity
WBC: white blood cell
